# Deciphering predictive factors for choice of thrombopoietin receptor agonist, treatment free responses, and thrombotic events in immune thrombocytopenia

**DOI:** 10.1038/s41598-019-53209-y

**Published:** 2019-11-13

**Authors:** Maria L. Lozano, Maria E. Mingot-Castellano, María M. Perera, Isidro Jarque, Rosa M. Campos-Alvarez, Tomás J. González-López, Gonzalo Carreño-Tarragona, Nuria Bermejo, Maria F. Lopez-Fernandez, Aurora de Andrés, David Valcarcel, Luis F. Casado-Montero, Maria T. Alvarez-Roman, María I. Orts, Silvana Novelli, Nuria Revilla, Jose R. González-Porras, Estefanía Bolaños, Manuel A. Rodríguez-López, Elisa Orna-Montero, Vicente Vicente

**Affiliations:** 1 Hospital Universitario Morales Meseguer, Centro Regional de Hemodonación, Universidad de Murcia, IMIB-Arrixaca, CB15/00055-CIBERER, Murcia, Spain; 2 Hospital Carlos Haya, Málaga; Hospital Universitario Virgen del Rocio, Sevilla, Spain; 3 Hospital Universitario de Gran Canaria Dr. Negrín, Las Palmas, Spain; 4 Hospital Universitario y Politécnico La Fe, Valencia, Spain; 5 Hospital de Especialidades de Jerez de la Frontera, Cádiz, Spain; 6 Hospital Universitario de Burgos, Burgos, Spain; 7 Hospital 12 de Octubre, Madrid, Spain; 8 Hospital San Pedro de Alcántara, Cáceres, Spain; 9 Complejo Hospitalario Universitario de A Coruña, A Coruña, Spain; 10 Complexo Hospitalario Universitario de Santiago, A Coruña, Spain; 11 Vall d’Hebron Institute of Oncology (VHIO), University Hospital Vall d’Hebron, Barcelona, Spain; 12 Hospital Virgen de la Salud, Toledo, Spain; 13 Hospital Universitario La Paz-Idipaz, Madrid, Spain; 14 Hospital de Sagunto, Valencia, Spain; 15 Hospital Sant Creu i Sant Pau, Barcelona, Spain; 16 Hospital Universitario Morales Meseguer, Murcia, Spain; 17 Hospital Universitario de Salamanca (HUSAL/IBSAL), and IBMCC (USAL-CSIC), Salamanca, Spain; 18 Hospital Clínico San Carlos, Madrid, Spain; 19 Hospital Álvaro Cunqueiro, Pontevedra, Spain; 20 Institut Català d’Oncologia-Hospital Germans Trias i Pujol, Badalona, Spain

**Keywords:** Haematological diseases, Immunological disorders

## Abstract

Very few data exist on when a particular thrombopoietin-receptor agonist (TPO-RA) is favored in clinical practice for the treatment of patients with immune thrombocytopenia (ITP), about novel risk factors for vascular events (VE) with these drugs, nor about predictive factors for therapy free responses (TFR). We conducted an observational, retrospective, long-term follow-up multicenter study from November 2016 to January 2018 of 121 adult ITP patients initiating TPO-RA between January 2012 to December 2014. Data reflected that a platelet count ≤25 × 10^9^/l at the time when the TPO-RA was initiated was associated with a 2.8 higher probability of receiving romiplostim *vs*. eltrombopag (*P* = 0.010). VE on TPO-RA was related to previous neoplasia in patients over 65 years (50% *vs*. 2.2%, *P* < 0.001), and to previous splenectomy in younger patients (100% *vs*. 33%, *P* = 0.001). Receiving romiplostim as first TPO-RA with no subsequent TPO-RA switching was associated with a 50% likelihood of TFR after 2.9 years of therapy (3.3 years in chronic ITP patients). These real-world data help deciphering some areas of uncertainty, and offer insight into some of the most relevant challenges of ITP which may help clinicians make appropriate treatment decisions in the management of adult ITP patients with TPO-RA.

## Introduction

Primary immune thrombocytopenia (ITP), is a rare acquired disease (ORPHA:3002) characterized by multiple alterations of the immune system leading to a significant reduction in the platelet number as a consequence of increased platelet destruction and impaired megakaryocytopoiesis^1^. The availability over the last decade of thrombopoietin receptor agonists (TPO-RA) for the treatment of ITP has transformed the management of this disease. Romiplostim, eltrombopag, and the recently FDA-licensed avatrombopag are TPO-RA that increase platelet counts in ITP by activating the c-mpl receptor, promoting the survival, proliferation, and differentiation of megakaryocytes and subsequently stimulating platelet production. Although many physicians are well-acquainted with these agents, very few data exist related to certain decision-making determinations.

These drugs, specifically romiplostim and eltrombopag, are recommended second-line therapy for adult ITP patients in guidelines recently updated^2,3^. This recommendation is the result of systematic evidence validated by randomized, controlled trials with a placebo comparator^4–6^. Although the two TPO-RA may have similar overall efficacy, randomized clinical trials comparing both drugs have not been performed. For that reason, caution is needed due to heterogeneity between the study designs, patient populations, and response definitions when interpreting the results of indirect treatment comparisons; one metaanalysis supports equivalent efficacy and safety^7^, while others suggest that romiplostim may have greater efficacy^8,9^. Since it is not possible to draw definite conclusions based on current evidence, the choice of which TPO-RA to use seems to be an individual one, based on physicians and patients preferences.

Despite the fact that 60–90% of patients will respond to the initial TPO-RA^10^, some patients do not show that beneficial effect. In that case, given that these agents have different pharmacodynamic and pharmacokinetic properties, switching to the alternative TPO-RA may be a suitable approach^11^. In clinical practice, tapering off TPO-RA is contemplated to assess if this therapy is no longer needed due to a decrease in the disease activity favoring sustained treatment-free responses (TFR). These TFR, which have been observed in up to 10–30% of ITP patients receiving these drugs^10^, raise the question of additional potential immunomodulatory properties by increasing the regulatory T and B cell compartment and decreasing platelet destruction^12,13^. To date, there are no predictors to identify in which patients this approach is likely to be successful, other than earlier start of TPO-RA (which may relate to the pathophysiology of the disease), and robust platelet responses^14–16^.

These agents have been proven to be well-tolerated, and adverse events are usually mild. Some reports indicate that TPO-RA have a potential for enhancing the inherent thrombotic risk of the disease (both venous and arterial). Although no properly designed trials have been conducted, the annualized thrombosis rates in adults with TPO-RA treatment appear to be 2–3 times higher than in an ITP population not treated with these agents^17^. Thromboembolic events appear to be more frequent in patients of older age and having at least 1 general risk factor for thrombosis, whereas it has no apparent relation to platelet count nor to the average or actual dose of either drug^17^. However, little information is available about different presentations of vascular events (VE) related to the TPO-RA being administered, or about the potential involvement of variables independent of traditional risk factors for thrombosis.

With the assumption that knowing additional post-authorization data about the use of TPO-RA can help to more strategically align the clinical practice and the management recommendations of patients with ITP, we undertook the present study aimed to (i) analyze practice patterns on the use of TPO-RA in “real-world” settings, with respect to indications, safety, switching, and TFR, and (ii) evaluate patients characteristics that might influence the selection of a particular TPO-RA over the other.

## Patients and Methods

### Study design and selection criteria

This was an observational, retrospective, multicenter study conducted in Spain from November 2016 to January 2018. The study was approved by the Clinical Research Ethics Committee of the Hospital General Universitario Morales Meseguer (Murcia, Spain) and the research project was carried out in accordance with the Declaration of Helsinki^18^. Written informed consent was obtained from every subject.

The participant investigators from 19 institutions with a wide geographic distribution had to recruit through the screening of the clinical records, all adult ITP patients who were alive and had started in their respective site TPO-RA from January 2012 to December 2014 as indefinite therapy. Patients were excluded if they had secondary ITP or if at the time they were prescribed the TPO-RAs they were participating in a clinical trial with investigational drugs.

The following information was obtained from the medical records: (1) demographics: age and sex; (2) diagnosis-related data: date of diagnosis, complete blood count (CBC), bleeding history, comorbidities, and diagnostic procedures performed; (3) previous therapies and responses; (4) TPO-RA administration: date and CBC at commencement, phase of the disease (newly diagnosed, persistent, or chronic ITP), bleeding, previous and concomitant treatment, indication for the TPO-RA, treatment duration, whether treatment with TPO-RA was switched and, if so, reason for switching, whether treatment with TPO-RA was discontinued by the time the information was recorded and, if so, reason for discontinuation, and achievement and duration of TFR. We considered TFR as platelet counts ≥50 × 10^9^/l for at least 6 months in the absence of any therapies meant to increase platelet counts. Bleeding severity was evaluated at the time of diagnosis and also when TPO-RA was initiated, with an ITP bleeding score that includes the severity of bleeding at 11 specific sites, with scores ranging from 0 to 2 (0 = none, 1 = mild, 2 = severe bleeding)^19^. Need for unscheduled hospital care requirements (emergency treatment or hospital admission) 6 months before and also 6 months after TPO-RA, and adverse events were also recorded. The final analysis was performed on data pooled from patients included from the 19 participating centers, with eleven institutions contributing three fourths of the total number of study participants.

### Statistical analysis

A descriptive statistics analysis, including median, ranges, and percentages, was performed. Differences in dichotomous variables were analyzed by Pearson’s χ^2^ test, and differences in continuous variables by the Mann-Whitney non-parametric test. Univariate logistic regression analyses were built in search of factors influencing odds ratios (OR) with 95% confidence interval (95% CI) of outcomes. Kaplan–Meier curves were built for TFR status within the study period. Results (given as percentages for categorical data, and median and range for continuous data) were considered statistically significant when the *P*‐value was less than 0.05. Statistical analysis was performed using SPSS software, version 23 (SPSS Inc., Chicago, IL).

## Results

A total of 121 patients (58.7% of them female) were enrolled in the study. In all cases patients were prescribed TPO-RA (romiplostim, n = 54; eltrombopag, n = 67) as indefinite therapy. The most frequent reasons for starting long-term treatment with TPO-RA were refractoriness or loss of response (n = 76, 62.8%) and the need for continuous administration of other drugs (n = 31, 25.6%). Other causes were the need to increase platelet counts due to antiplatelet or anticoagulant therapy (n = 4, 3.3%), or the wish to delay splenectomy (n = 9, 7.4%). Baseline characteristics of the study cohort are presented in Table 1. When TPO-RA were initiated, the majority of cases (67.8%) were chronic ITP (median time from diagnosis 65.6 months; 12.7 to 603.4 months), while 15.7% of patients had persistent and 16.5% newly diagnosed disease. The median initial doses of the eltrombopag were 350 mg/week (150–525 mg/week), and 2 μg/kg/week (1–7 μg/kg/week) in the case of romiplostim. To capture enough events to reveal meaningful patterns, a long-term follow-up study was designed. The median time from start of TPO-RA until collection of data was of 44.9 months (23.8 to 67.5 months), whereas that under TPO-RA treatment was 35.2 months (1 to 67.3 months). Although the initial intention to treat was indefinite therapy, at last follow-up only 65 patients (53.7%) continued to receive treatment with TPO-RA. There was a trend towards using eltrombopag in older patients (median 66 years; 21–96 years, romiplostim median age 57 years; 19–90 years; *P* = 0.115), and with more comorbid conditions, although differences were not statistically significant except for hypertension (Table 1). Neither gender, liver disease, phase of the disease, previous history of neoplasia or VE, nor exposure or responses to previous lines of therapy were associated with the choice of TPO-RA (*P* > 0.05) (Table 1).

**Table 1:** Patients’ characteristics and previous therapies.

	All (N = 121)	Romiplostim (N = 54)	Eltrombopag (N = 67)	*P*
Gender (% Female)	58.7	59.3	58.2	0.907
Platelets at diagnosis (median, range)	11.5, 0–95	12, 1–82	11, 0–95	0.899
Age (median, range)	63, 19–96	57, 19–90	66, 21–96	0.115
Hypertension (%)	28.1	16.7	37.3	0.012
Diabetes (%)	14.0	11.1	16.4	0.404
Liver disease (%)	4.1	3.7	4.5	0.832
Previous neoplasia (%)	6.6	5.5	8.1	0.675
Previous vascular event (%)	9.1	7.4	7.2	0.563
Chronic phase (%)	67.8	68.5	67.2	0.874
Patients that started TPO-RA in the first 18 months (January 2012–June 2013) (%)	48.8	53.7	44.8	0.329
**Previous therapies**				
1^st^ line Prednisone (%) • Responders (%)	85.9 65.4	90.7 69.4	82.1 61.8	0.173 0.335
Dexamethasone (%) • Responders (%)	29.7 61.1	33.3 55.5	26.9 66.7	0.439 0.494
Rituximab (%) • Responders (%)	19.0 56.5	25.9 71.4	13.4 33.3	0.082 0.072
Splenectomy (%) • Responders (%)	17.1 62.9	18 61.1	17 64.7	0.337 0.826
IVIG (%) • Responders (%)	57.8 78.6	59.3 81.2	56.7 76.3	0.778 0.616
Immunosuppressants (%) • Responders (%)	13.2 31.2	13.0 28.6	13.4 33.3	0.979 0.838
Danazol (%) • Responders (%)	10.7 46.1	13.0 42.9	8.9 50.0	0.479 0.797

Immunosuppresants include azathioprine, cyclophosphamide, or cyclosporine.

We assessed whether the prescription of a particular TPO-RA could be related with the risk of bleeding^19^. At diagnosis of the disease, bleeding symptoms were more frequent and more severe among patients that were subsequently treated with romiplostim than with eltrombopag (66.7% *vs*. 41.8% of bleeders, *P* = 0.006; median cumulative bleeding score of 2 *vs*. 0, *P* = 0.003). At the time when TPO-RA was initiated, bleeding manifestations and platelet counts were also correlated with the selection of TPO-RA; the median cumulative bleeding score was of 1 *vs*. 0, *P* = 0.037, and the median platelet counts of 18 × 10^9^/l *vs*. 25 × 10^9^/l, *P* = 0.014, in romiplostim and eltrombopag treated patients, respectively. Additionally, six months before the start of TPO-RA, the requirement for hospital care (emergency treatment or hospital admission) was higher in patients that would later on receive romiplostim than in those elected for eltrombopag (55.6% vs. 31.8%, *P* = 0.009) (Table 2). Among the 74 episodes of unscheduled hospital visits, 75.7% of the cases were related to bleeding, and 12.2% to infection. In 62.2% of the episodes where patients presented at the hospital, they were discharged following management in the emergency department (in 83.9% due to hemorrhagic symptoms), and non-bleeding causes accounted for 35.7% of the admissions to hospitals.

**Table 2:** Univariate logistic regression model for factors related to bleeding risk potentially affecting the selection of a particular TPO-RA. OR indicates the probability of choosing romiplostim over eltrombopag.

	OR	95% CI	*P*
Bleeding at diagnosis	2.7	1.29–5.73	**0.009**
Bleeding score at diagnosis >2	2.9	1.25–6.57	**0.013**
Bleeding score when TPO-RA was started >1	2.5	1.18–5.46	**0.017**
Platelet count when TPO-RA was started ≤25 × 10^9^/l	2.8	1.27–6.04	**0.010**
Need for unscheduled hospital care in the previous 6 months	2.7	1.27–5.74	**0.010**

Abbreviations: TPO-RA, thrombopoietin receptor agonist.

### Safety. Thrombotic events

We examined whether the initiation of TPO-RA had an impact on the patient need for unscheduled hospital care requirements. The use of TPO-RA was associated with a significant reduction in the percentage of patients, and in the number of times these patients required emergency treatment or hospital admission in the 6 months following- compared to the 6 months before- TPO-RA therapy (20.2% *vs*. 42%, P = 0.007; 37 *vs*. 74 episodes, P = 0.031).

The tolerance of the agents was generally very good; adverse events were usually mild, most frequently causing myalgia, arthralgia, headache, or abdominal discomfort, and reported at equally low rates (<10%) in patients with either eltrombopag or romiplostim. One patient receiving romiplostim was diagnosed with a solid tumor, and in two cases a significant increase in liver enzymes was reported with eltrombopag. Nine individuals in each group underwent bone marrow assessment following TPO-RA, and bone marrow fibrosis was reported in two patients under romiplostim therapy. In one patient bone marrow examination was not repeated, whereas in the other, one year after romiplostim was stopped a regression of myelofibrosis from from MF-3 to MF-1 was confirmed. The assessment of the rate of VE during the interval under treatment of 329.3 patient-year revealed 15 patients experiencing 17 VE. These included 9 thrombotic events (6 pulmonary embolism, 3 venous thrombosis), and 8 ischemic episodes (3 stroke, 2 transient ischemic attack, 1 angina pectoris, 1 myocardial infarction, 1 peripheral ischemic event). TPO-RA were continued during the acute event in 10 cases, and later resumed in 4 additional episodes where patients were transitorily switched to a different ITP therapy. In the course of venous thrombosis, anticoagulation was always initiated, and antiplatelet therapy was started in all patients presenting with ischemia. In all cases but one (sequela of mild dysarthria), the clinical recovery from the VE was complete.

Before TPO-RA, 11 of the 121 included patients had presented VE; nonetheless TPO-RA did not seem to predispose to new events, since such history of prior thromboembolic or ischemic events was present only in one of the 15 patients with VE during TPO-RA therapy. The median platelet count when the VE under TPO-RA occurred was 110 × 10^9^/l (10–648 × 10^9^/l). Ten out of the 15 patients continued TPO-RA at last follow-up. Seven events occurred with romiplostim, and 10 with eltrombopag. The annualized risk was 4.2 and 5.9 VE/100 patient-years in romiplostim and eltrombopag treated patients, respectively (median 5.2). The calculated incidence therefore was of one VE for every 17 patient-year receiving eltrombopag, and one VE event for every 23 patient-year receiving romiplostim. Most VE occurred in the first year of TPO-RA therapy (median 276 days; range 5–1183), with a trend toward earlier events under romiplostim than eltrombopag (127 days *vs*. 360 days, respectively; *P* = 0.070). In the case of ischemic events the median time to arterial events was 165 *vs*. 606 days in romiplostim and eltrombopag treated patients; *P* = 0.029.

The comorbidities of patients that subsequently developed VE included one patient presenting with antiphospholipid antibodies, five having been diagnosed with neoplasia, one with vascular peripheral disease, and two with hypothyroidism –one of whom also had renal disease. When the TPO-RA was started, all patients were reported to have sustained complete remission of previous neoplasias (1 colon carcinoma; 1 Burkitt lymphoma; 1 bladder cancer; 1 breast cancer; 1 patient with history of thyroid, breast and ovarian tumors) and none of them was receiving any cancer-specific therapies. There were no significant differences in the 15 patients *vs*. the 106 patients that did not suffer from VE in terms of gender, age, diabetes, hypertension, previous vascular events, or time on prednisone as 1st line therapy. Factors associated with the occurrence of a VE were previous splenectomy (53.3% *vs*. 25.5%, *P* = 0.026), chronic phase of the disease (93.3% *vs*. 64.1%, *P* = 0.024), and a personal history of malignancy (33.0% *vs*. 2.8%, *P* < 0.001). Table 3 shows the results of the univariate logistic regression model. Splenectomy and previous malignancy were found to be significant factors related to thrombotic events. However, when patients were dichotomized according to age, just in those over 65 years experiencing VE on TPO-RA (8 out of 54) a history of previous neoplasia was associated with VE (50% *vs*. 2.2%, *P* < 0.001), whereas only in younger patients with VE during TPO-RA therapy (7 out of 67), previous splenectomy was related to those incidents (100% *vs*. 33%, *P* = 0.001).

**Table 3:** Univariate logistic regression model for factors related to vascular events during TPO-RA therapy. OR indicates the probability of having a vascular event under TPO-RA therapy in the presence of a specific factor.

	OR	95% CI	*P*
**Univariate analysis**			
Splenectomy	3.3	1.11–10.09	0.032
Chronic disease	7.8	0.99–61.83	0.051
Previous malignancy	17.2	3.57–82.65	<0.001

Abbreviations: TPO-RA, thrombopoietin receptor agonist.

### Switching and discontinuation. Therapy free responses

Although initially more patients received eltrombopag than romiplostim as chronic therapies (n = 67 *vs*. n = 54, respectively), due to switching, the exposure to both TPO-RAs was similar: 80 patients received romiplostim for a total exposure of 161.0 years, and also 80 individuals were treated with eltrombopag for total exposure of 168.3 years. Overall, 39 patients (32.2%) switched TPO-RA, with a trend towards more common drug exchange in eltrombopag-treated patients (38.8% of those that initiated this TPO-RA) than among romiplostim-treated patients (24.1% of patients that initially received this agent) (*P* = 0.085). The rates of TPO-RA switching were not significantly different between chronic ITP patients and those with non-chronic phases (35.4% vs. 25.6%, respectively; P = 0.285). The reasons for switching differed between the initial TPO-RA being administered; efficacy issues were more frequent among patients treated with eltrombopag (17 out of the 26 patients that switched eltrombopag *vs*. 2 out of 13 that switched romiplostim; *P* = 0.003), while patients’ or doctors’ preference was a more common cause to substitute romiplostim (8 patients) than eltrombopag (2 patients), *P* < 0.001. Other reasons for switching included adverse events (5 patients with eltrombopag), platelet fluctuation (2 cases with romiplostim), or perioperative surgical procedures (1 with romiplostim, and 2 with eltrombopag).

**Figure 1:**
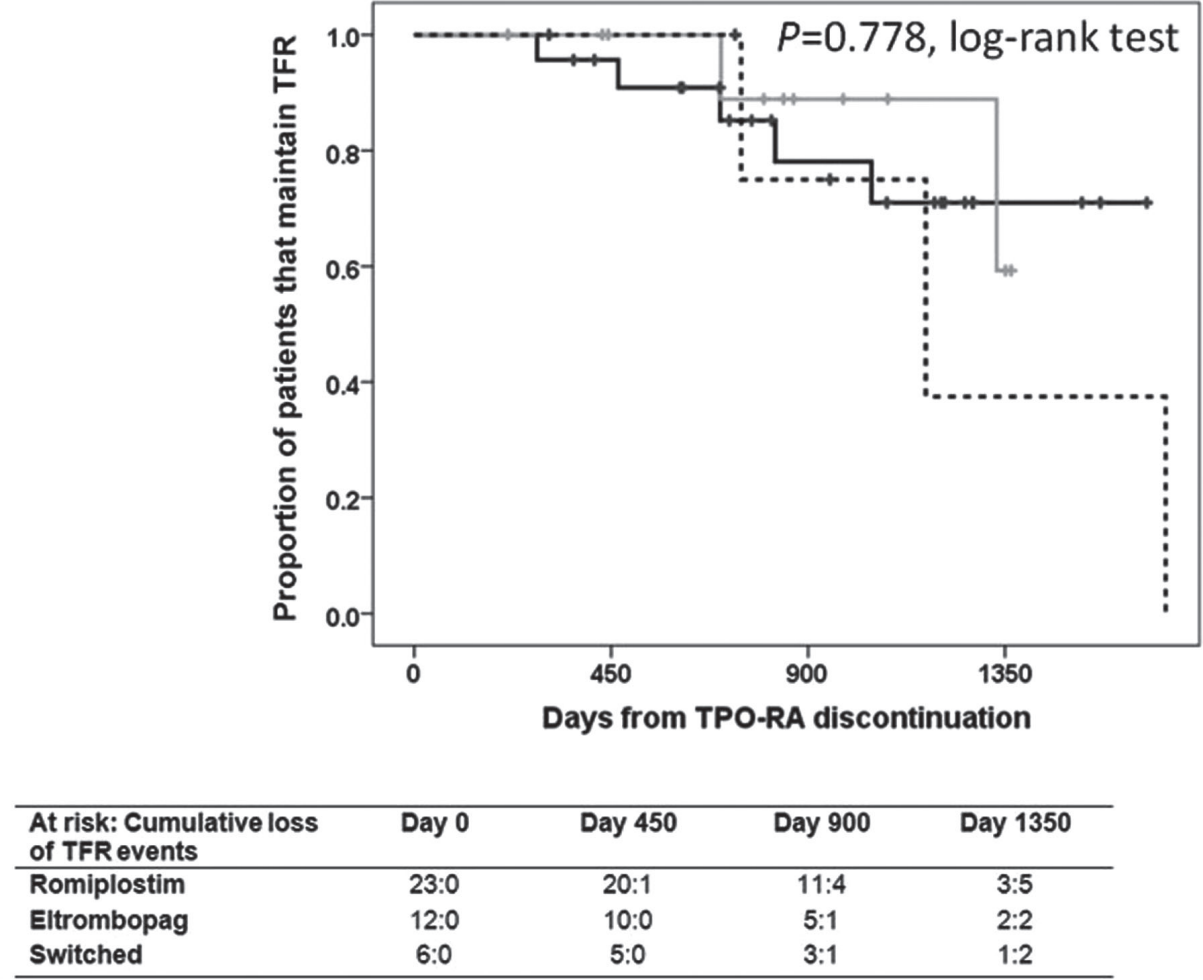
Probability of maintaining therapy free responses (TFR) upon TPO-RA discontinuation. Kaplan-Meier plot showing the estimated probability of TFR in patients who discontinue TPO-RA for reasons other than lack of efficacy and being followed for a minimum of 6 months (n = 41). Patients who died while on TPO-RA therapy were not included in the study. Solid black line represents patients that received only romiplostim (n = 23); solid grey line represents patients that received only eltrombopag (n = 12), and dashed black line represents patients that switched TPO-RA (n = 6). The number of patients that discontinue TPO therapy (“at risk”) and the cumulative loss of TFR events at time points are presented for each group below the figure.

From the start of TPO-RA until data collection almost one half of the patients (46.3%, n = 56) tapered-off TPO-RA. Patients that discontinued TPO-RA non due to lack of response and with a follow-up longer than 6 months (n = 41), were followed for 27.1 months (9.2–55.0) in the case of romiplostim (n = 23), 28.1 months (7.0–44.8) for those that stopped eltrombopag (n = 12), 20.7 months (10.1–31.2) for patients tapering off romiplostim having previously received eltrombopag (n = 2), and 31.5 months (24.1–56.4) for those ceasing eltrombopag having switched from romiplostim (n = 4). The Kaplan Meier curve of relapse-free remission of these 41 patients is displayed in Fig. 1. A total of 35 patients (85.4% of those that stopped TPO-RA) achieved TFR after a median time under TPO-RA treatment of 14.2 months. Romiplostim was discontinued in 21 cases, eltrombopag in 10 cases, and in 4 cases patients received both TPO-RA due to switching. In 4 of these 35 patients (3 patients having received romiplostim, and 1 having switched TPO-RA), these agents were reintroduced due to loss of response after a median TFR period of 20.5 months.

Potential predictors of TFR in the remaining 31 patients (25.6%) that sustained platelet counts for a median of 32 months (8–217 months) were analyzed. No specific patient features (e.g. age, comorbidities, ITP duration, previous treatments, bleeding, nor phase of disease) seemed to consistently predict for TFR (*P* > 0.05). However, statistically significant predictors of TFR were identified: interestingly, while the specific TPO-RA that was discontinued did not influence the probability to achieve TFR, romiplostim as first TPO-RA was positively associated (3.0 increased probability) with TFR (*P* = 0.011), while switching TPO-RA was associated with a 8.8 risk of not achieving TFR (*P* = 0.004) (Table 4).

**Table 4:** Univariate logistic regression model for factors related to therapy free responses. OR indicates the probability of therapy free responses associated to the factor indicated.

	OR	95% CI	*P*
Romiplostim as first TPO-RA	3.0	1.28–7.01	**0.011**
No switching	8.8	1.97–39.26	**0.004**

Abbreviations: TPO-RA, thrombopoietin receptor agonist.

Although there was a significant difference in the initial response to the first TPO-RA (7% *vs*. 25% of non-responders to romiplostim and eltrombopag, respectively; *P* = 0.009), however, other additional factors to platelet responses should be involved in the different TFR between TPO-RA. Data revealed that in patients that received only romiplostim (n = 41), or solely eltrombopag (n = 41), the rate of responses were similar (95.1% responders in both groups not exposed to switching). In contrast, the probability of attaining TFR in patients that received only romiplostim was 3.2 higher than those exposed only to eltrombopag (*P* = 0.014).

Of the 41 patients that were treated with romiplostim (median time 26.1 months; 1.0–67.3), 21 of them (51.3%) achieved TFR. When considering the 41 patients treated with eltrombopag (median time on TPO-RA of 38.6 months; 1.5–63.8), ten of them (24.4%) were able to discontinue the drug successfully. The percentage of patients that maintained TFR after tapering off TPO-RA was 15.4% of those switching from romiplostim to eltrombopag, and 7.7% of those that alternated from eltrombopag to romiplostim (median duration of TPO-RA therapy of 44.2 months; 4.4–61.6, and 34.1 months; 2.4–61.4, respectively).

To minimize the confounding variable of mistakenly attributing TPO-RA induced TFR, when in fact it could be a consequence of the natural course of the underlying disease, we analyzed the subset of 82 patients with chronic ITP (median time from diagnosis of ITP to therapy with TPO-RA of 5.5 years; 1.1–50.3 years). In this group of patients, there was a trend towards receiving romiplostim as first TPO-RA and attaining sustained responses (P = 0.073), while switching TPO-RA was significantly associated with a 6.4 risk of not achieving TFR (P = 0.019). In chronic ITP patients, TFR were achieved in 44.8% of patients after romiplostim discontinuation, and in 25% following tapering off eltrombopag; TFR were observed in 12.5% among those that alternated from romiplostim to eltrombopag, and in 4.7% of patients switching from eltrombopag to romiplostim.

**Figure 2:**
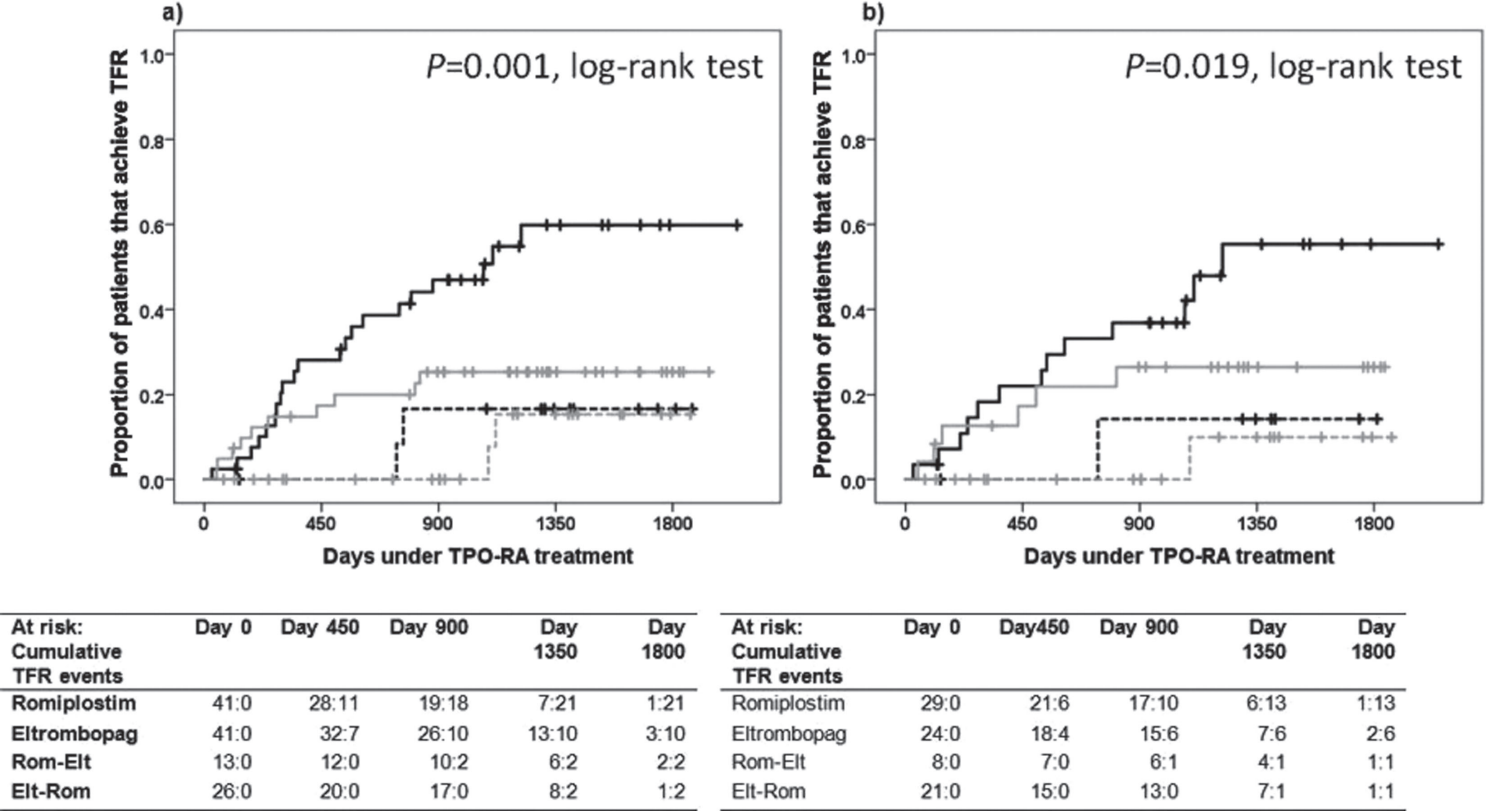
Probability of achieving therapy free responses (TFR). Proportion of patients achieving TFR within the whole cohort (n = 121) included in the study (panel 2a), and in those with chronic ITP (panel 2b). TFR was defined as the ability of a patient to discontinue TPO-RA as platelet counts >50 × 10^9^/l for at least 6 months in the absence of any therapies meant to increase platelet counts. Patients who died while on TPO-RA therapy were not included in the study. Solid black line represents patients that received only romiplostim (Panel a, n = 41; Panel b, n = 29). Solid grey line represents patients that received only eltrombopag (Panel a, n = 41; Panel b, n = 24). Dashed black line represents patients that initiated romiplostim and switched to eltrombopag (Panel a, n = 13; Panel b, n = 8). Dashed grey line represents patients that initiated eltrombopag and switched to romiplostim (Panel a, n = 26; Panel b, n = 21). The number of patients under TPO therapy (“at risk”) and the cumulative TFR at time points are presented for each group below each figure.

Data reflected that patients receiving romiplostim and not experiencing switching were the best performing group in terms of achieving TFR. Thus, in the whole cohort, 50% of patients tapered off the drug after a median of 2.9 yrs (95% CI 1.7–4.2 years) and sustained TFR (Fig. 2a), while the same behavior was seen in chronic ITP patients after a median of 3.3 years (95% CI 2.7–4.0 years) (Fig. 2b).

## Discussion

Eltrombopag and romiplostim are effective drugs that have been established as a mainstay in the treatment of patients with ITP providing an excellent long-term outlook. Response rates from clinical trials^7–9^ and clinical practice data^20^ do not seem to differ greatly between both agents, though none of the studies provided a randomized comparison. Thus, the choice between eltrombopag and romiplostim is a matter of availability, convenience and cost. Our observational data indicate that in real world setting, the choice of one TPO-RA over the other for long-term treatment of ITP could be conditioned by attributes other than the mode of administration of these drugs and/or patient’s preference. We found that lower platelet counts and hemorrhagic symptoms relate to a higher likelihood of prescribing romiplostim over eltrombopag. This suggests that the prescription of the TPO-RA could be related to some extent with the risk of bleeding before initiating treatment. In these situations, romiplostim is given preferentially to patients, probably based on the wider dosage range of the drug that may allow earlier responses with higher doses, as suggested in a small sample size study^21^. Additionally, the subcutaneous administration avoids potential dietary interferences, and adherence to treatment is secured. In agreement with our clinical practice data, a recent network analysis indicates that romiplostim may rank slightly higher than eltrombopag for the probability of achieving early responses^9^. However, due to the limitations of the few randomized clinical trials that were included, inaccurate assumptions should not be made until reliable data demonstrate distinct response patterns with different TPO-RA.

Similar to the above mentioned paucity information related to time to platelet responses, data about differences in the overall platelet responses with these agents is also scarce, with some studies reporting similar outcomes^7^, and others indicating that romiplostim produces better results^8,9^. In our cohort of patients, lack of response as reason for switching TPO-RA was more frequent among eltrombopag-treated patients (25.4% *vs*. 3.7% of those initially treated with each drug). These results differ from more than 250 reported cases that have been switched from one TPO-RA to another^11^. The analysis of patients included in 12 studies reflects that in total 67/103 (eltrombopag to romiplostim) and 76/165 (romiplostim to eltrombopag) switched for non-efficacy issues^22,23^. The incongruence of the current and previous data may be based on (i) the heterogeneity in management of patients receiving or not maximum product dose as per prescribing information (eltrombopag 75 mg/day, or romiplostim 10 μg/kg, in both cases for 4 weeks) prior to TPO-RA switching, which may influence efficacy, and (ii) previous reports do not express data as percentage of patients that alternate among those that start a specific TPO-RA, so that results cannot be directly compared.

In general, the tolerability of these drugs was very good, and adverse events were not a major reason for switching or for discontinuation. In fact, these therapies were associated with decreased healthcare resource utilization defined as hospital admission or emergency department visits. Similar to other reports^17,24^, TPO-RA however seemed to increase the incidence of thrombosis, with an annualized risk of 5.2 VE/100 patient-years in TPO-RA treated patients. Interestingly, we found that the history of curable malignancy was a strong factor associated with the occurrence of thromboembolic or ischemic events in elderly ITP patients. It is well-known that active cancer is associated with a 4- to 7-fold increased risk of venous thromboembolism compared with non-cancer patients^25,26^, probably due to factors related to cancer per se (e.g. a hypercoagulable state, or treatment-related effects)^27^. Limited data suggest that these mechanisms resulting in an increased risk of VE in cancer may persist even after the cancer is in remission^28^. In line with that, in ITP patients, a past history of malignancy increases the risk of venous thromboembolism with an HR of of 1.8^29^. However, in our study the risk of VE in ITP patients with a history of previous neoplasia while on TPO-RA was disproportional (more than 40-fold increased risk in elderly patients), suggesting a novel association of these variables with VE in elderly ITP patients under TPO-RA therapy. Our data indicate that however, at younger ages, splenectomy is the strongest factor influencing the likelihood to suffer from these events. Although further research is warranted, these data highlight the relevance of the evaluation of age and comorbidities before initiation of TPO-RA, including the history of malignancies and splenectomy, when managing the care of these patients.

In the last years there has been progress in our knowledge of potential TFR in a number of patients treated with TPO-RA, and such data from the published series, together with the good efficacy/safety profile have positioned these drugs as one of the second-line treatments of choice today, as confirmed by recent guidelines^2,3^. Information is still sparse regarding prognostic factors and their implications for treatment. In particular, we designed a study with long-term assessment of outcomes with TPO-RA, to better enable the evaluation of outcomes of therapies with these agents. Additionally, we aimed to assess clinical predictors of TFR by dealing with confounding variables, specifically minimizing the possible bias of attributing TPO-RA induced TFR, when in fact it may be the natural course of disease, by analyzing a group of chronic ITP patients with a particularly poor baseline prognosis (median time from diagnosis 5.5 years).

In this long-term follow-up analysis, we confirm that platelet responses following TPO-RA cessation is sustained in 25.6% of adult patients with primary ITP, and identified variables that in real life seem to predict significantly higher odds of TFR. While in our study the specific TPO-RA that was discontinued did not influence the probability to achieve TFR, receiving romiplostim as first TPO-RA was positively associated with a 3-fold greater likelihood of achieving TFR, while switching TPO-RA negatively predicted sustained platelet responses. The fact that the percentage of responders was similar in patients that received only a single type of TPO-RA indicates that factors other than platelet responses may be involved in the increased TFR observed in romiplostim treated individuals. Whether this is due to the recently proposed hypothesis of tolerogenic properties of romiplostim, attributed to the “carrying” Fc molecule^30^ or to additional factors, is still an unanswered question to be unveiled.

Receiving romiplostim as first TPO-RA and avoiding its switching associates with a 50% likelihood of TFR after 2.9 years of therapy. These data did not differ substantially from those of chronic ITP patients, where assuring that long duration under TPO-RA therapy was provided (median of 3.3 years), one half of chronic ITP patients treated with romiplostim and not undergoing switching also achieved TFR.

The main limitation of our study is its observational retrospective design, although it could also represent a strength, since it encompasses heterogeneous patients in the clinical practice managed by different doctors in distinct institutions. From these data we conclude that in clinical practice, TPO-RAs are frequently used as second-line therapies, regardless of the chronicity of the disease. In this context, there seems to be a preferential use of romiplostim for patients with increased bleeding risk, which also appears to associate with higher TFR, while both TPO-RA exhibit a similar tolerability and safety profile. However, we acknowledge that results of our study are difficult to put into perspective with previous observational studies because of differences in populations, follow-up and outcomes. For that reason, a long-term pragmatic randomized clinical trial would be desirable to overcome some the above-mentioned limitations and to corroborate these findings.

## Conclusion

This retrospective multicenter study with long-term follow-up offers insight into some of the most relevant real-world challenges in the treatment of ITP patients with TPO-RA. Data reflect that in clinical practice, the patient’s hemorrhagic risk determines the choice TPO-RA, and distinguishes novel variables associated with vascular events under therapies with thrombopoietic agents. Additionally, we confirm that more than one-fourth of patients can discontinue these agents and sustain TFR, and identify predictive clinical markers that indicate that one half of specifically managed subsets of ITP patients can achieve these TFR.

## Data Availability

The datasets used and/or analyzed during the current study are available from the corresponding author on reasonable request.
