# Author Correction: Deciphering predictive factors for choice of thrombopoietin receptor agonist, treatment free responses, and thrombotic events in immune thrombocytopenia

**DOI:** 10.1038/s41598-021-95732-x

**Published:** 2021-08-13

**Authors:** Maria L. Lozano, Maria E. Mingot-Castellano, María M. Perera, Isidro Jarque, Rosa M. Campos-Alvarez, Tomás J. González-López, Gonzalo Carreño-Tarragona, Nuria Bermejo, Maria F. Lopez-Fernandez, Aurora de Andrés, David Valcarcel, Luis F. Casado-Montero, Maria T. Alvarez-Roman, María I. Orts, Silvana Novelli, Nuria Revilla, Jose R. González-Porras, Estefanía Bolaños, Manuel A. Rodríguez-López, Elisa Orna-Montero, Vicente Vicente

**Affiliations:** 1 Hospital Universitario Morales Meseguer, Centro Regional de Hemodonación, Universidad de Murcia, IMIB-Arrixaca, CB15/00055-CIBERER, Murcia, Spain; 2 Hospital Carlos HayaMálaga; Hospital Universitario Virgen del Rocio, Sevilla, Spain; 3 Hospital Universitario de Gran Canaria Dr. Negrín, Las Palmas, Spain; 4 Hospital Universitario y Politécnico La Fe, Valencia, Spain; 5 Hospital de Especialidades de Jerez de la Frontera, Cádiz, Spain; 6 Hospital Universitario de Burgos, Burgos, Spain; 7 Hospital 12 de Octubre, Madrid, Spain; 8 Hospital San Pedro de Alcántara, Cáceres, Spain; 9 Complejo Hospitalario Universitario de A Coruña, A Coruña, Spain; 10 Complexo Hospitalario Universitario de Santiago, A Coruña, Spain; 11 Vall d’Hebron Institute of Oncology (VHIO), University Hospital Vall d’Hebron, Barcelona, Spain; 12 Hospital Virgen de la Salud, Toledo, Spain; 13 Hospital Universitario La Paz-Idipaz, Madrid, Spain; 14 Hospital de Sagunto, Valencia, Spain; 15 Hospital Sant Creu i Sant Pau, Barcelona, Spain; 16 Hospital Universitario Morales Meseguer, Murcia, Spain; 17 Hospital Universitario de Salamanca (HUSAL/IBSAL), and IBMCC (USAL-CSIC), Salamanca, Spain; 18 Hospital Clínico San Carlos, Madrid, Spain; 19 Hospital Álvaro Cunqueiro, Pontevedra, Spain; 20 Institut Català d’Oncologia-Hospital Germans Trias i Pujol, Badalona, Spain

Correction to: *Scientific Reports* https://doi.org/10.1038/s41598-019-53209-y, published online 13 November 2019

The original version of this Article contained errors in Figures 1 and 2.

**Figure 1:**
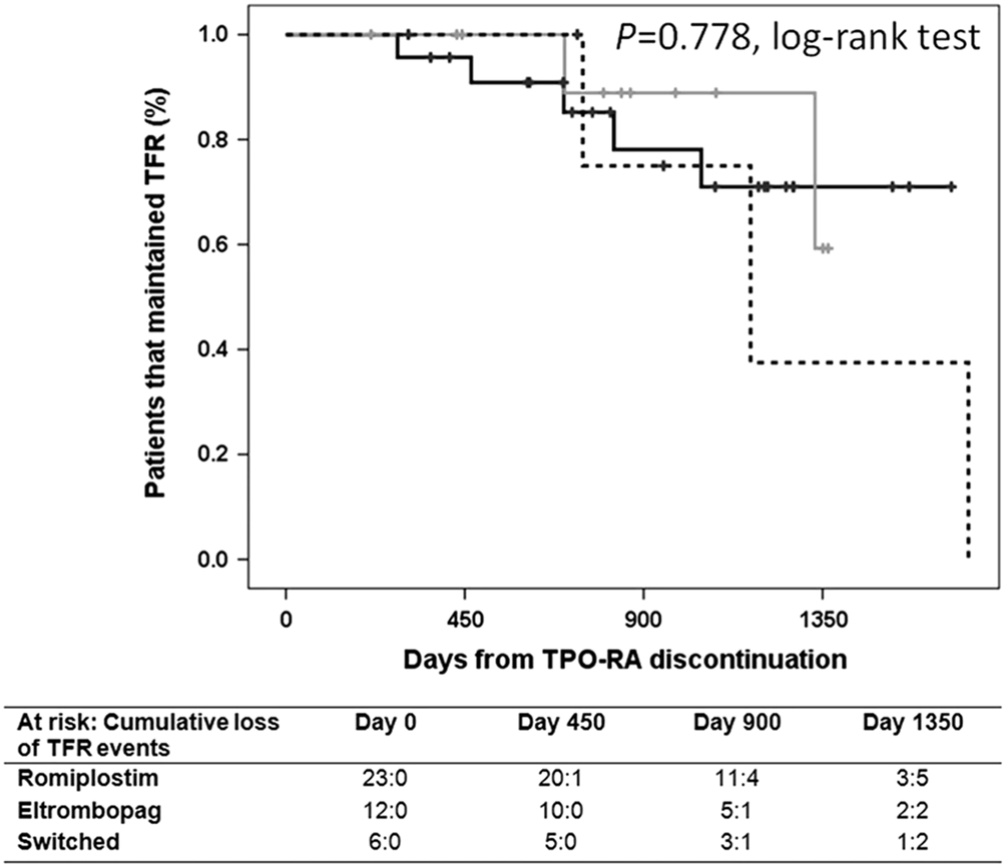
Probability of maintaining therapy free responses (TFR) upon TPO-RA discontinuation. Kaplan–Meier plot showing the estimated probability of TFR in patients who discontinue TPO-RA for reasons other than lack of efficacy and being followed for a minimum of 6 months (n = 41). Patients who died while on TPO-RA therapy were not included in the study. Solid black line represents patients that received only romiplostim (n = 23); solid grey line represents patients that received only eltrombopag (n = 12), and dashed black line represents patients that switched TPO-RA (n = 6). The number of patients that discontinue TPO therapy (“at risk”) and the cumulative loss of TFR events at time points are presented for each group below the figure.

**Figure 2:**
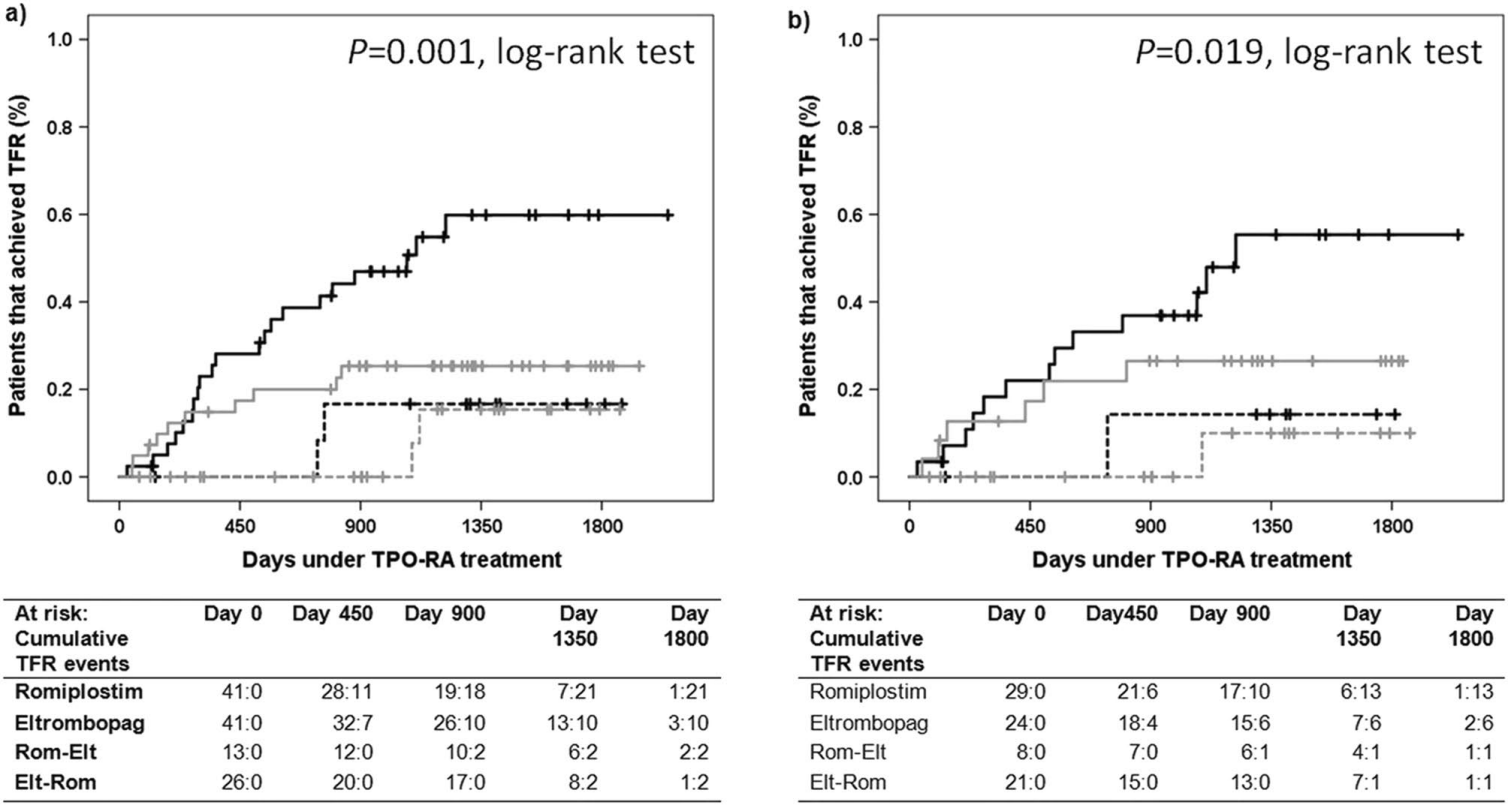
Probability of achieving therapy free responses (TFR). Proportion of patients achieving TFR within the whole cohort (n = 121) included in the study (panel **a**), and in those with chronic ITP (panel **b**). TFR was defined as the ability of a patient to discontinue TPO-RA as platelet counts > 50 × 10^9^/l for at least 6 months in the absence of any therapies meant to increase platelet counts. Patients who died while on TPO-RA therapy were not included in the study. Solid black line represents patients that received only romiplostim (Panel **a**, n = 41; Panel **b**, n = 29). Solid grey line represents patients that received only eltrombopag (Panel **a**, n = 41; Panel **b**, n = 24). Dashed black line represents patients that initiated romiplostim and switched to eltrombopag (Panel **a**, n = 13; Panel **b**, n = 8). Dashed grey line represents patients that initiated eltrombopag and switched to romiplostim (Panel **a**, n = 26; Panel **b**, n = 21). The number of patients under TPO therapy (“at risk”) and the cumulative TFR at time points are presented for each group below each figure.

In Figure 1, the y-axis label, “Proportion of patients that maintain TFR” was incorrectly given as “Patients that maintained TFR (%)”. In Figure 2, the y-axis label, “Proportion of patients that achieve TFR” was incorrectly given as “Patients that achieved TFR (%)”. The original Figures 1 and 2 and accompanying legends appear below.

The original Article has been corrected.

